# An Outpatient, Ambulant-Design, Controlled Human Infection Model Using Escalating Doses of *Salmonella* Typhi Challenge Delivered in Sodium Bicarbonate Solution

**DOI:** 10.1093/cid/ciu078

**Published:** 2014-02-10

**Authors:** Claire S. Waddington, Thomas C. Darton, Claire Jones, Kathryn Haworth, Anna Peters, Tessa John, Ben A. V. Thompson, Simon A. Kerridge, Robert A. Kingsley, Liqing Zhou, Kathryn E. Holt, Ly-Mee Yu, Stephen Lockhart, Jeremy J. Farrar, Marcelo B. Sztein, Gordon Dougan, Brian Angus, Myron M. Levine, Andrew J. Pollard

**Affiliations:** 1 OxfordVaccine Group, Department of Paediatrics, University of Oxford; 2 National Institute for Health Research Oxford Biomedical Research Centre; 3 Microbial Pathogenesis Group, Wellcome Trust Sanger Institute, Hinxton, United Kingdom; 4 Department of Biochemistry and Molecular Biology and Bio21 Molecular Science and Biotechnology Institute, University of Melbourne, Victoria, Australia; 5 Centre for Statistics in Medicine, University of Oxford; 6 Emergent Product Development UK, Emergent BioSolutions, Wokingham, United Kingdom; 7 Oxford University Clinical Research Unit, Hospital for Tropical Diseases, Ho Chi Minh City, Viet Nam; 8 Nuffield Department of Medicine, University of Oxford, United Kingdom; 9 Center for Vaccine Development, University of Maryland School of Medicine, Baltimore

**Keywords:** typhoid fever, *Salmonella* Typhi, enteric infection, controlled human infection, human challenge study

## Abstract

***Background.*** Typhoid fever is a major global health problem, the control of which is hindered by lack of a suitable animal model in which to study *Salmonella* Typhi infection. Until 1974, a human challenge model advanced understanding of typhoid and was used in vaccine development. We set out to establish a new human challenge model and ascertain the *S.* Typhi (Quailes strain) inoculum required for an attack rate of 60%–75% in typhoid-naive volunteers when ingested with sodium bicarbonate solution.

***Methods.*** Groups of healthy consenting adults ingested escalating dose levels of *S.* Typhi and were closely monitored in an outpatient setting for 2 weeks. Antibiotic treatment was initiated if typhoid diagnosis occurred (temperature ≥38°C sustained ≥12 hours or bacteremia) or at day 14 in those remaining untreated.

***Results.*** Two dose levels (10^3^ or 10^4^ colony-forming units) were required to achieve the primary objective, resulting in attack rates of 55% (11/20) or 65% (13/20), respectively. Challenge was well tolerated; 4 of 40 participants fulfilled prespecified criteria for severe infection. Most diagnoses (87.5%) were confirmed by blood culture, and asymptomatic bacteremia and stool shedding of *S.* Typhi was also observed. Participants who developed typhoid infection demonstrated serological responses to flagellin and lipopolysaccharide antigens by day 14; however, no anti-Vi antibody responses were detected.

***Conclusions.*** Human challenge with a small inoculum of virulent *S.* Typhi administered in bicarbonate solution can be performed safely using an ambulant-model design to advance understanding of host–pathogen interactions and immunity. This model should expedite development of diagnostics, vaccines, and therapeutics for typhoid control.

## Abstract (precis)

Oral delivery of escalating-dose *Salmonella* Typhi (Quailes strain) using sodium bicarbonate buffer solution in an outpatient, ambulant-design human infection study demonstrates safety, requires a lower challenge inoculum than that used in historical studies, and offers a unique insight into host–pathogen interactions.

Typhoid infection is a major global health problem [1]. *Salmonella enterica* serovar Typhi (*S*. Typhi) is a human-restricted pathogen; in the absence of suitable animal models, many details of the human response to infection remain unclear [2, 3]. From 1952 to 1974, human typhoid challenge studies were performed at the University of Maryland to address this critical issue [2, 4–6]. A successful model was developed in which volunteers ingested *S*. Typhi suspended in 45 mL of milk; without buffer, 50% of participants developed clinical infection following ingestion of 10^7^ colony-forming units (CFU) of wild-type *S.* Typhi [2, 5]. Data from this model were directly applied to improve understanding of antibiotic mechanisms [7] and used in vaccine (a first step toward eventual licensure of Ty21a vaccine) [8–10] and diagnostic development [11, 12].

Here we describe the development of a new controlled human infection model of *S.* Typhi challenge using outpatient, ambulant participants. The primary objective of this study was to ascertain the challenge inoculum (“dose”) of *S.* Typhi (Quailes strain) required to produce an attack rate of 60%–75% in typhoid-naive volunteers when ingested with sodium bicarbonate buffer solution.

## METHODS

### Study Design

**Figure 1.**
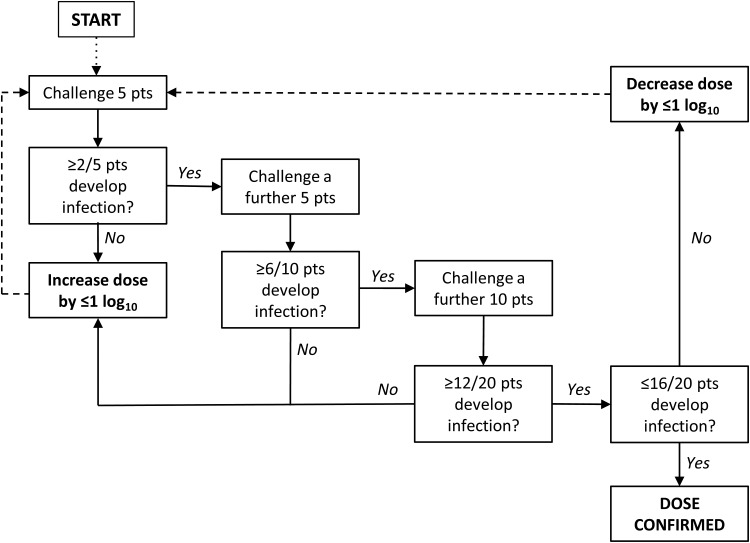
Dose-escalation decision algorithm. Abbreviation: pts, patients.

An observational, dose-escalation study of controlled human infection using *S.* Typhi (Quailes strain) was performed. The challenge agent was delivered by oral ingestion of bacteria suspended in sodium bicarbonate solution (NaHCO_3_[aq]) using a predetermined dose-escalation strategy (Figure 1). Attack rate was defined as the proportion of participants diagnosed with infection by day 14 after challenge meeting clinical (temperature ≥38°C sustained for ≥12 hours) and/or microbiological (blood culture–confirmed *S*. Typhi bacteremia) endpoints (per protocol population; “typhoid diagnosis”). An independent data and safety monitoring committee reviewed participant safety and attack rate data throughout the study, in particular cumulative data gathered following the first, fifth, 10th, and 20th challenges performed. Secondary objectives included description of the human response to and the microbiological dynamics of infection.

The study was approved by Oxfordshire Research Ethics Committee A (10/H0604/53) and conducted in accordance with the principles of the International Conference of Harmonisation Good Clinical Practice guidelines.

### Setting and Participants

The study was performed at the Centre for Clinical Vaccinology and Tropical Medicine, Oxford, United Kingdom. The United Kingdom is nonendemic for typhoid fever; the rate of typhoid fever notification in Oxfordshire is 0.5 per 100 000, the majority of which is travel related [13].

Healthy adults aged 18–60 years who had not previously received typhoid vaccination or been resident >6 months in typhoid-endemic areas were eligible to participate. With written informed consent, screening was performed to assess participant health status. Exclusion criteria were extensive and included any significant medical, surgical, or psychiatric illness; evidence of gallbladder disease (by ultrasound examination); antibiotic allergy; food-handling occupation; or contact with susceptible individuals (including healthcare workers and carers of young children or elderly or immunocompromised persons). Consent was not obtained from close household contacts, but participants were required to provide these contacts with written information providing details regarding the study and measures to reduce the risk of infection. After completion of antibiotics, participant contacts were also offered the opportunity to be screened for infection.

### Challenge Strain

*Salmonella* Typhi Quailes strain was isolated in 1958 from the gallbladder of a known chronic carrier. Quailes strain can express the Vi antigen and is fully antibiotic susceptible [2, 5]. A fresh working cell bank was manufactured under Good Manufacturing Practices (GMP) guidelines prior to storage at −80°C. Whole-genome sequencing was used to determine the phylogenetic relationship of the Quailes strain to other *S.* Typhi (Supplementary Figure 1). These data also confirmed that the challenge strain encoded the expected repertoire of virulence-associated determinants.

### Inocula and Challenge

Inocula were freshly prepared prior to each challenge by defrosting and suspending the required number of bacteria in 30 mL/0.53 g NaHCO_3_(aq). Participants fasted for 90 minutes before ingesting 120 mL/2.1 g NaHCO_3_(aq). Two minutes later, participants ingested the prepared challenge suspension and were monitored for 15 minutes. To calculate the actual challenge dose given, direct plating of aliquots from the challenge inoculum and remaining working cell bank vials was performed in triplicate using tryptone soya agar (Oxoid) and culture for 24 hours before colony counting.

### Clinical Evaluation of Participants

Participants were reviewed daily for at least 14 days, recording the duration and severity of all solicited (Supplementary Table 1) and unsolicited symptoms experienced, and twice-daily oral temperature readings, using supplied diary cards. Participants had 24-hour access to a study physician; additional reviews were performed if infection was suspected, at medical discretion, and at participant request.

Indications for antibiotic treatment (ciprofloxacin, 500 mg twice daily, 14 days) included reaching typhoid diagnosis, unmanageable symptoms, or clinical necessity. All remaining participants received treatment at day 14. Treatment compliance was ensured by direct observation of antibiotic ingestion at study visits and daily reminders by telephone contact and/or text message.

Subsequent visits were performed at 21, 28, and 60 days after challenge. Clearance of infection after treatment was confirmed by bacterial culture of ≥2 stool specimens obtained at least 1 week apart, at least 3 weeks after completion of antibiotics.

### Assessment of Hematological, Biochemical, and Serological Response to Challenge

Routine blood hematology and biochemistry were assessed on alternate days after challenge and at typhoid diagnosis; a maximum of 1110 mL blood was collected from each study participant by day 28. Serological response to challenge was performed using blood collected at baseline (day 0) and 14, 28, and 60 days later. Specific immunoglobulin G (IgG), immunoglobulin M (IgM), and immunoglobulin A (IgA) responses to Vi polysaccharide (Sanofi Pasteur MSD, Maidenhead), lipopolysaccharide (LPS) (L2387; Sigma-Aldrich, Dorset), and flagellin (prepared in-house by shearing and centrifugation of a whole-cell preparation) were measured in serum by enzyme-linked immunosorbent assay, using goat antihuman IgG, IgM, and IgA conjugated to specific horseradish peroxidase (AbD Serotec, UK).

### Assessment of Microbiological Dynamics After Challenge

*Salmonella* Typhi shedding in stool was assessed using daily self-collected samples, cultured according to national standard operating procedures [14]. Blood (10 mL or 5 mL at typhoid diagnosis) was cultured daily by direct inoculation into broth (BACTEC Plus Aerobic vials, BD) and subsequent automated growth detection (BACTEC FX System, BD), in accordance with standard methods [15]. *Salmonella* Typhi growth and serotype were confirmed by biochemical profile (API-10S, bioMérieux, France) or slide agglutination according to the Kauffman-White classification, respectively [16].

Quantitative blood culture was performed at typhoid diagnosis by inoculation of 10 mL blood into an ISOLATOR 10 tube (a commercial lysis-centrifugation system; Alere, UK). After centrifugation, the resulting pellet was directly plated onto XLD agar (Oxoid, UK). Quantitative stool culture was performed by suspending 1 g of stool in sodium selenite, followed by subculturing onto XLD agar (Oxoid). Blood- or stool-inoculated plates were then incubated (37°C for 24 hours) prior to identification and counting.

### Statistical Analyses

An initial challenge dose of 10^3^CFU was chosen as the highest dose from the historical studies that did not lead to clinical infection [5, 17, 18]. With a final sample size of 20 participants at each dose level, 95% confidence intervals (CIs) of 36%–81% and 46%–88% for measured attack rates of 60% or 70%, respectively, were anticipated. Analyses are descriptive and comprise proportions or means with associated 95% CIs. Diagnostic odds ratios (DORs) (a single indicator describing the ratio of the odds of positivity in infection relative to the odds of positivity in the noninfected; 1 indicating that the test does not discriminate between infected and uninfected) [19] and 95% CIs were calculated to assess performance of individual symptoms reported for typhoid diagnosis to be made.

Clinical, laboratory, microbiological, and immunological data were collated using a Web-interface database (OpenClinica Community, version 2.1) and Microsoft Excel (2010 edition). Data analysis was performed using SPSS (version 16.0, IBM SPSS) and GraphPad Prism (version 5, GraphPad, Inc).

## RESULTS

### Participants

**Figure 2.**
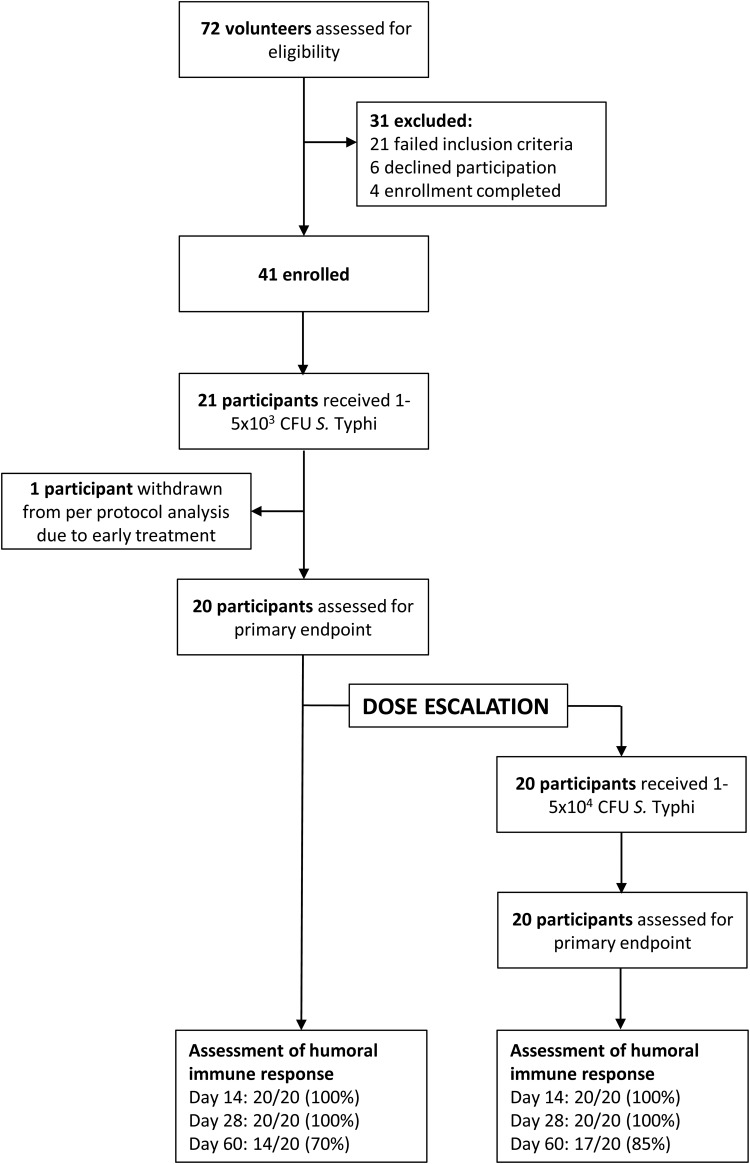
Participant recruitment, enrollment, and disposition flow diagram for clinical study OVG 2009/10. Abbreviations: CFU, colony-forming units; *S.* Typhi, *Salmonella enterica* serovar Typhi.

From February to October 2011, 41 participants were enrolled and challenged with 10^3^ or 10^4^ CFU of *S.* Typhi (Table 1). Forty participants were included in the per protocol analysis (Figure 2); 1 participant challenged with 10^3^ CFU was treated for symptoms related to infection before day 14 without meeting diagnostic criteria and was therefore excluded.

**Table 1.** Participant Demographic Characteristics and Challenge Dose Data

Characteristic	Dose 1	Dose 2	All
Target range *S.* Typhi challenge dose	1–5 × 10^3^ CFU	10–50 × 10^3^ CFU	…
No. of participants challenged (per protocol analysis)	21 (20)	20 (20)	41 (40)
Actual *S.* Typhi challenge dose, median [IQR]			
All participants	1.34 × 10^3^ CFU [0.98–1.69]	19.8 × 10^3^ CFU [18.8–21.6]	…
Typhoid diagnosed subsequently	1.05 × 10^3^ CFU [0.97–1.58]	20.3 × 10^3^ CFU [18.8–20.3)	…
Typhoid not diagnosed subsequently	1.39 × 10^3^ CFU [1.00–1.79]	19.4 × 10^3^ CFU [18.8–22.8]	…
Sex, male, No. (%)	17 (81)	12 (60)	29 (71)
Age, y, mean ± SD (range)	29.8 ± 9.4 (19.4–46.5)	30.2 ± 8.8 (19.6–45.1)	30.0 ± 9.0 (19.4–46.5)
Ethnicity, white, No. (%)	20 (95)	19 (95)	39 (95)
Tobacco smoking, yes, No. (%)	5 (24)	8 (40)	13 (32)
Alcohol intake, units^a^, median [IQR]	6 [3–10]	5 [1.25–10]	6 [2–10]

Abbreviations: CFU, colony-forming units; IQR, interquartile range; *S.* Typhi, *Salmonella enterica* serovar Typhi; SD, standard deviation.

^a^ 1 unit = 10 mL or 8 g pure alcohol equivalent consumed per week.

### Clinical Safety

Challenge was well tolerated; no participants required hospital admission, intravenous antibiotics, or fluids. Four participants fulfilled criteria for severe infection (Table 2). No episodes of shedding or carriage following the initiation of antibiotics were identified.

**Table 2.** Attack Rates and Incubation Periods for the Per Protocol Population

	Target Challenge Dose	
Outcome Measure	10^3^ CFU (n = 20)	10^4^ CFU (n = 20)
Predefined diagnostic criteria reached for typhoid diagnosis, No. (%)		
Clinical (temperature) with blood culture confirmation	6 (30)	5 (25)
Clinical (temperature) only	1 (5)	2 (10)
Blood culture with clinical signs of typhoid fever	1 (5)	5 (25)
Blood culture only	3 (15)	1 (5)
Overall attack rate, No. (%)	11 (55)	13 (65)
Severe typhoid fever, No. (%)		
Oral temperature ≥40°C	0 (0)	2 (10)
Grade 3 or higher laboratory abnormality	1 (5)^a^	1 (5)^b^
All severe typhoid diagnoses, No. (%)	1 (5)	3 (15)
Incubation periods, d, mean ± SD (No.), median [IQR]		
Time to initiation of antibiotic treatment	11.65 ± 3.27 (20) 14 [8.75–14]	9.9 ± 3.35 (20) 9 [4–14]
Typhoid-diagnosed participants only, d, mean ± SD, (No.), median [IQR]		
Time to microbiological and/or clinical diagnosis	9.73 ± 3.35 (11) 9 [6.5–13]	7.69 ± 1.65 (13) 8 [6–9]
Time to clinical diagnosis	7.57 ± 1.74 (7) 7.5 [6–8.75]	7.57 ± 1.90 (7) 7 [6–8.5]
Time to microbiological diagnosis	13.5 ± 0.58 (4) 13.5 [13–14]	7.83 ± 1.72 (6) 8 [6.25–9]
Attack rates with alternative diagnostic criteria, all participants^c^, No. (%)		
Oral temperature measurement ≥37.5°C (any duration)	11 (52)	14 (70)
Oral temperature measurement ≥38°C (any duration)	10 (48)	13 (65)
Oral temperature measurement ≥38.5°C (any duration)	8 (38)	11 (55)
Oral temperature measurement ≥38°C (any duration) or bacteremia	12 (57)	14 (70)
Oral temperature measurement ≥38°C (any duration) and bacteremia	7 (33)	10 (50)
Oral temperature measurement ≥38°C (any duration) and subsequent bacteremia	5 (24)	8 (40)
Oral temperature measurement ≥38°C (any duration) and subsequent bacteremia OR positive stool culture	7 (33)	10 (50)
Oral temperature measurement ≥38°C (any duration) OR bacteremia OR positive stool culture	14 (67)	14 (70)
Symptoms		
Average No. of solicited symptoms reported per participant, mean ± SD	20.4 ± 15.9	27.6 ± 2.8
Participants reporting at least 1 severe symptom, grade 3 or higher, No (%)	8 (40)	10 (50)

Predefined criteria for typhoid diagnosis were either clinical (reaching and sustaining an oral temperature of ≥38°C for ≥12 hours) or microbiological (having 1 or more confirmed positive blood cultures). Criteria for severe typhoid included any of the following: oral temperature measurement ≥40°C, lethargy or confusion, gastrointestinal bleeding or perforation, or any grade 3 or higher laboratory abnormality (these are described for the 2 individual cases in footnotes a and b below).

Abbreviations: CFU, colony-forming units; IQR, interquartile range; SD, standard deviation.

^a^ Increase in alanine aminotransferase to 10 times the upper limit of normal (45 IU/L): day 10, 90 IU/L; day 12, 483 IU/L; day 14, 667 IU/L; day 31, 46 IU/L.

^b^ Hypokalemia (<3.1 mmol/L): day 6, 3.5 mmol/L; day 7, 3.1 mmol/L; day 8, 3.0 mmol/L; day 12, 3.8 mmol/L.

^c^ Including the participant excluded from the per protocol analysis.

### Attack Rates

Typhoid diagnosis was made in 11 of 20 (55% [95% CI, 32%–77%]) participants challenged with 10^3^ CFU of *S.* Typhi. Dose escalation to 10^4^ CFU resulted in 13 of 20 (65% [95% CI, 41%–84%]) participants developing infection (Table 1). Ten participants (4 in the 10^3^ CFU group and 6 in the 10^4^ CFU group) were diagnosed based on blood culture result alone, of whom 4 remained persistently afebrile (temperature <38°C). Two participants recorded temperatures ≥38°C and reported mild symptoms but remained “undiagnosed,” as fever was not sustained for 12 hours and all blood cultures remained negative.

### Clinical Response

**Figure 3.**
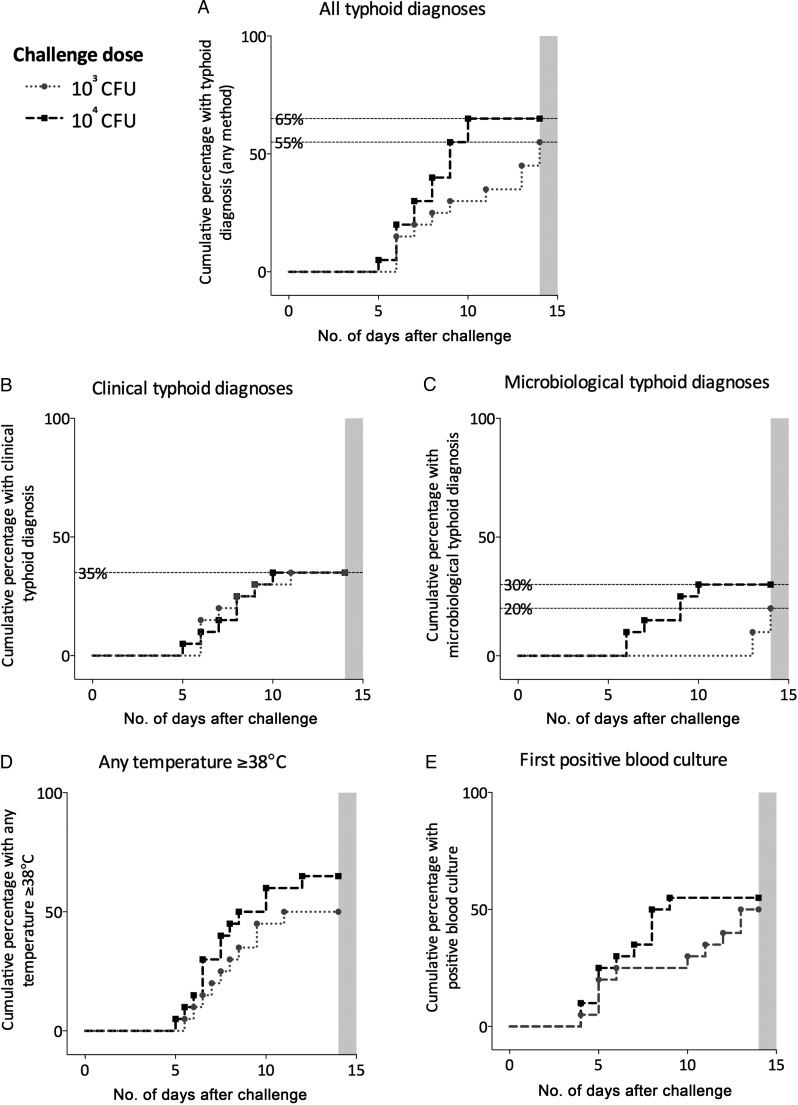
Kaplan-Meier plots demonstrating time to diagnostic endpoints including all diagnoses (*A*), those diagnosed using clinical (*B*) and microbiological (*C*) criteria, and time to fever (first temperature ≥38°C; [*D*]) and to first positive blood culture (*E*), following challenge at 2 dose levels (10^3^ or 10^4^ colony-forming units [CFU]) of *Salmonella* Typhi (Quailes strain). Shaded gray area, end of 2-week observation period (all remaining participants started on antibiotic therapy); dotted gray line with circle symbols, participants challenged with 10^3^ CFU; dashed black lines with square symbols, participants challenged with 10^4^ CFU.

Overall median interval from challenge to initiation of antibiotic treatment was 12 days (10^3^ CFU, 14 days; 10^4^ CFU, 9 days; Table 2, Figure 3*A*). In those participants developing infection, the median incubation period from challenge to diagnosis was 8 days (10^3^ CFU, 9 days [interquartile range {IQR}, 6.5–13]; 10^4^ CFU, 8 days [IQR, 6–9]). In participants fulfilling clinical diagnostic criteria, no difference was seen in time from challenge to diagnosis (Table 2, Figure 3*B*).

Symptoms consistent with typhoid infection were reported from day 4. The clinical profile of infection varied considerably between participants, although headache was universally reported by those infected (DOR, 23.4 [95% CI, 1.2–460.7]; Supplementary Figure 2 and Supplementary Table 1). Arthralgia was the most discriminatory symptom (DOR, 57.0 [95% CI, 6.0–541.5]; Supplementary Figure 2*C*).

Clinical observations made are summarized in Table 3. Relative bradycardia was seen at typhoid diagnosis in 4 of 7 participants with temperatures ≥38.3°C (ie, recorded heart rate <110 bpm) or 2 of 2 participants with temperatures ≥38.9°C (heart rate <120 bpm) [20].

**Table 3.** Overview of Clinical Findings

Clinical Finding	Target Challenge Dose	
	10^3^ CFU (n = 20)	10^4^ CFU (n = 20)
Radial pulse, bpm, median [IQR]		
Screening visit	70 [64–73.5]	64 [58.5–78.5]
Baseline (day 0)	70.5 [63.8–78]	71 [58.8–83.8]
Typhoid diagnosis	91 [80.5–103.5]	95 [87–102]
Day 14	77 [72.3–80]	78.5 [70.3–86.8]
Systolic blood pressure, mm Hg, median [IQR]		
Screening visit	126 [119–139]	127 [119–136]
Baseline (day 0)	122 [117–137]	121 [113–139]
Typhoid diagnosis	125 [123–140]^a^	124 [120–130]^b^
Day 14	125 [118–135]	121 [115–131]
Oral temperature, °C, median [IQR]		
Baseline (day 0)	36.3 [36.1–36.4]	36.3 [36.1–36.6]
Typhoid diagnosis	37.9 [37–38.6]	37.8 [37.4–38.1]
Day 14	36.2 [36–36.5]	36.3 [36–36.5]
Rash, No. (%)^c^	4 (20)	4 (20)

Abbreviations: bpm, beats per minute; CFU, colony-forming units; IQR, interquartile range; mm Hg, millimeters of mercury.

^a^ Relative bradycardia in 2 of 7 participants with temperature ≥38.9°C (heart rate [HR] <120 bpm) and 3 of 7 participants with temperature ≥38.3°C (HR <110 bpm).

^b^ Relative bradycardia in 0 of 7 participants with temperature ≥38.9°C (HR <120 bpm) and 1 of 7 participants with temperature ≥38.3°C (HR <110 bpm).

^c^ Any rash recorded between challenge and day 21. One of 4 and 2 of 4 participants recording a rash after challenge with 10^3^ or 10^4^CFU, respectively, were diagnosed with typhoid infection.

### Hematological and Biochemical Response

In participants developing typhoid infection, laboratory abnormalities were evident prior to the onset of clinical typhoid symptoms (Supplementary Figure 3).

Hemoglobin concentrations fell progressively in all groups after challenge (Supplementary Figure 3*E*). An initial fall in eosinophil fractions was evident in participants subsequently developing infection (Supplementary Figure 3*I*). Total white cell count remained within the normal range until diagnosis, at which point total white cell count and neutrophil and lymphocyte fractions dropped to low levels (Supplementary Figure 3*G*, *H*, and *J*). Thrombocytopenia was seen in all participants diagnosed with typhoid infection; platelet counts fell to <150 × 10^3^ cells/µL in 11 of 24 participants (Supplementary Figure 3*F*).

### Microbiological Dynamics

*Salmonella* Typhi was cultured from blood in 10 of 11 (90.9%) and 11 of 13 (84.6%) diagnosed participants given 10^3^ or 10^4^ CFU, respectively (Table 4); however, those participants ingesting 10^4^CFU had a greater proportion of positive blood cultures prior to antibiotics being initiated (10^3^ CFU, 6.2%; 10^4^ CFU, 9.6%). Quantitative blood culture performed at typhoid diagnosis demonstrated median bacterial loads of 0.47 CFU/mL and 1.10 CFU/mL in those challenged with 10^3^ CFU and 10^4^CFU, respectively (Table 4).

**Table 4.** Microbiological Dynamics of Bacterial Shedding and Bacteremia in Study Participants

Microbiological Measure	10^3^CFU Dose			10^4^CFU Dose		
	Typhoid Diagnosed	Typhoid Not Diagnosed	All	Typhoid Diagnosed	Typhoid Not Diagnosed	All
Blood cultures						
* S.* Typhi–positive cultures, No. (%)	19/305 (6.2)	…	…	34/353 (9.6)	…	…
* *Bacteremic participants, No. (%)	10/11 (90.9)	…	…	11/13 (84.6)	…	…
* *Mean No. of *S.* Typhi–positive cultures per participant with typhoid diagnosis	1.7	…	…	2.6	…	…
* *Mean No. of *S.* Typhi–positive cultures per bacteremic participant	1.9	…	…	3.1	…	…
* *Quantitative culture (all performed), CFU/mL, median [IQR]	0.5 [0–1.2]	…	…	1.1 [0.4–2.1]	…	…
* *Quantitative culture (positive cultures only), CFU/mL, median [IQR]	1.1 [0.5–1.4]	…	…	1.5 [0.9–2.4]	…	…
Stool cultures						
* S.* Typhi–positive cultures (any time point), No. (%)	22/151 (14.6)	2/116 (1.7)	24/267 (11.2)	29/178 (16.3)	1/96 (1.0)	30/274 (10.9)
* S.* Typhi–positive cultures (prior to 72 h postchallenge), No. (%)	5/22 (15)	1/17 (6)	6/39 (15.1)	6/21 (29)	1/13 (8)	7/34 (20.6)

Abbreviations: CFU, colony-forming units; IQR, interquartile range; *S.* Typhi, *Salmonella enterica* serovar Typhi.

Early shedding of *S.* Typhi (detected within 72 hours of challenge) was found in 30% of all participants, of whom 10 of 12 (83%) subsequently developed infection (DOR, 5 [95% CI, .9–27.1]). *Salmonella* Typhi was subsequently shed by 18 of 24 (75%) of typhoid-diagnosed participants; shedding preceded typhoid diagnosis in 12 of 18 participants. One otherwise asymptomatic participant (10^3^ CFU dose) had *S.* Typhi cultured from stool specimens taken 13 days after challenge. Stool quantification data for participants challenged with the 10^3^ CFU dose demonstrated that the median number of bacteria excreted was 32 (IQR, 18–41) CFU/g feces (n = 13).

### Serological Response

Low levels of anti-H and anti-Vi antibody were measured in all participants at baseline (Supplementary Figure 4 and Supplementary Table 2), and did not correlate with subsequent risk of infection using the study diagnosis definition (data not shown). In participants developing typhoid infection, increases in IgG, IgM, and IgA to LPS and H antigens were seen, whereas anti-Vi levels remained unchanged throughout (Supplementary Figure 4). Little change was seen in participants not succumbing to infection after challenge.

## DISCUSSION

This is the first investigation of the human pathophysiological response to *S.* Typhi challenge in nearly 40 years [2]. We have demonstrated that human challenge with *S.* Typhi (Quailes strain) using an outpatient model is safe and well tolerated, even in participants developing blood culture–confirmed symptomatic infection. Using coadministered sodium bicarbonate solution, an almost 4-log_10_ lower dose of *S.* Typhi was required to produce the target attack rate of 65% in comparison to historical challenge studies [5, 17]. A target attack rate of 60%–75% was selected based on historical typhoid challenge data from Maryland, providing a balance between using a smaller and therefore more physiological exposure dose while achieving a moderately high attack rate. A relatively high attack rate is advantageous in performing vaccine and treatment studies as smaller sample sizes are required, thereby reducing the number of individuals exposed and making studies more logistically feasible [18].

For some human challenge models of bacterial enteropathogens, eg, *Vibrio cholerae* O1, inocula require coadministration of a buffer agent for clinical infection to ensue. For others, such as *Shigella dysenteriae* or enterotoxigenic *Escherichia coli*, administration with sodium bicarbonate buffer both reduces the inoculum dose required and results in a more consistent pattern of clinical infection [21–24]. From this study, *Salmonella* Typhi challenge appears to fall into the latter category, the features of which are seen as desirable prerequisites for using a human challenge approach to assess vaccine and therapeutic efficacy. In particular, lower levels of pathogen exposure are thought to more closely replicate natural infection and there is less chance of overwhelming any infection- or vaccine-derived protective responses seen, an issue noted previously in typhoid challenge/vaccine studies [10, 22, 23, 25].

Diagnostic endpoint definitions are critical to determining the attack rate in human challenge studies, for drawing conclusions regarding dose response, and for their practical application to assessment of diagnostics, vaccines, and therapeutics. The effect of altering the stringency of the diagnostic criteria to make a diagnosis of typhoid infection was highlighted retrospectively in a reanalysis of the Maryland studies; the stringent criteria used potentially led to missed cases of milder disease, thus revealing a dose response when less stringent criteria were used [17]. We provide some confirmation of this dose response with use of bicarbonate, in which a higher challenge dose produces a higher attack rate (with a variety of definitions) and a shorter duration to the development of bacteremia. Interestingly, there was no association between dose and severity of clinical disease or time to onset of symptoms, which was also noted in the Maryland studies [26]. The reasons for this disparity remain unclear and are under further investigation. Sensitivity analysis around the diagnostic definition we used demonstrates a wide range of resulting attack rates, from 32% to 68% (Table 2). We suggest that reporting a range of outcomes in this way is useful for extrapolation to a variety of different settings; that is, a more relaxed endpoint definition may be most useful in calculating likely vaccine efficacy in field settings, whereas strict criteria may be more applicable to the development of novel diagnostic assays.

This study has also provided further data regarding the “milder” end of the typhoid infection spectrum, confirming that subclinical and asymptomatic infections may be relatively more common than previously anticipated or measured. Although cases of asymptomatic bacteremia were occasionally seen in the Maryland studies, these were often complicated by prior receipt of endotoxin challenge or may have been explained by previous vaccination or exposure [27]. Here, we demonstrate that asymptomatic bacteremia is a distinct pattern of infection after exposure (challenge), which may have implications for assessment of patients in the field, and for transmission modeling and assessment of vaccine mechanisms.

Whereas the range of symptoms reported by participants in our study is in keeping with those from previous Maryland studies and community settings, in general, our participants were more symptomatic throughout the challenge period [28–33]. Reasons for this may include heightened symptom reporting (and solicitation) due to perceived study risk profile, lower levels of preexisting immunity, or the relatively high exposure dose used in this artificial setting. This range and severity of symptoms may also be more in keeping with the true early, untreated (both antibiotic, analgesic, and anti-inflammatory) clinical presentation of typhoid infection, however [34, 35], and are more compatible with those noted among ill returning travellers, rather than patients in endemic settings or children [30–33].

Important laboratory findings in this study include further detail regarding the development of hematological changes prior to and following the development of clinical typhoid infection. A gradual fall in hemoglobin and hematocrit was seen in all participants, likely as a result of the venesections performed, but for which there may be additional explanations including bacterial suppression of bone marrow function (a fall in platelets was also seen in typhoid-diagnosed participants). Early and almost complete loss of eosinophils from peripheral blood was observed in all participants subsequently developing infection. There are few previous reports regarding this phenomenon, but these include observations made in returning travelers [36]; although not specific for typhoid fever diagnosis, this finding may be useful in predicting likely outcome in the highly controlled challenge setting.

Although the number of bacteria required to cause natural typhoid infection remains to be elucidated, microbiological blood quantification data provide indirect support for the doses used here. We found very low numbers of bacteria to be present in the peripheral blood at the time of clinical diagnosis, a finding supported by field data, albeit in an endemic setting [37]. These very low numbers reiterate the complexity of developing useful sensitive diagnostics for typhoid. Many participants were stool culture positive in the days preceding the development of bacteremia and fever, a period that did coincide with the initial onset of symptoms (headache, abdominal discomfort, etc). This prepatent period may represent a period of heightened transmission risk, for example, to household contacts caring for a sick relative, and supports renewed national recommendations for contact screening [38].

Longitudinal sample collection during the course of bacterial challenge provides unique insight into the kinetics of antibody response following pathogen exposure. In agreement with previous studies, we found relatively high baseline levels of *S.* Typhi–specific LPS antibody in nonexposed/nonvaccinated study participants [39]. Contrary to the findings from Maryland, these baseline titers did not correlate with subsequent infection risk. Similarly, those participants not developing typhoid fever consistently demonstrated scant response to the surface-expressed antigens studied [28]. Although the use of different antigen preparations in various studies may be partially responsible, the reasons for this discrepancy remain unclear. In participants diagnosed with typhoid, most developed measurable LPS and H antibody responses by day 14 or day 28; among those who did not were several individuals with blood culture–confirmed infection. This finding underscores a key central limitation in using serology to sensitively detect acute infection. Interestingly, no change in Vi antibody titer was seen in study participants, supporting previous findings from Maryland and those from studies of engineered Vi^+^ Ty21a vaccine strains [28, 40]. One reason for initially choosing the Quailes strain for challenge was its consistent Vi expression; indeed, all *S.* Typhi isolates retrieved from blood in this study demonstrated Vi positivity. Failure to mount an antibody response to the Vi antigen in acute infection has not been adequately explained and requires further investigation; these data do, however, suggest that natural infection–derived immunity is unlikely to be Vi mediated.

Limitations to the interpretation of data derived from this typhoid challenge model include logistic, ethical, and financial factors restricting the feasible challenge observation period to 2 weeks. The usual incubation period following exposure to natural infection is 7–14 days (range, 3–60 days) [41]; hence, treatment of all participants with antibiotics at day 14 may have curtailed or prevented infections that would otherwise have occurred had observation been extended. Furthermore, it is possible that the Quailes strain is not truly representative of currently circulating virulent strains; however, we demonstrate close phylogenetic relatedness to known disease-causing strains (Supplementary Figure 1).

In summary, we report the successful development of a new controlled human infection model of *S.* Typhi using healthy community volunteers. In addition to enabling detailed investigation of host–pathogen interactions and providing data to inform transmission modeling, use of this new challenge model will facilitate and expedite the development of new diagnostics, vaccines, and therapeutics, which are much needed for improved global control and ultimate eradication of this human blight.

## Supplementary Data

Supplementary materials are available at *Clinical Infectious Diseases* online (http://cid.oxfordjournals.org/). Supplementary materials consist of data provided by the author that are published to benefit the reader. The posted materials are not copyedited. The contents of all supplementary data are the sole responsibility of the authors. Questions or messages regarding errors should be addressed to the author.

Supplementary Data
