# Supplementary material for: An Outpatient, Ambulant-Design, Controlled Human Infection Model Using Escalating Doses of Salmonella Typhi Challenge Delivered in Sodium Bicarbonate Solution
Source: Clin Infect Dis. 2014 Feb 10;58(9):1230–40. doi: 10.1093/cid/ciu078 (PMC3982839; doi:10.1093/cid/ciu078)
Supplement: Supplementary Data [file supp_58_9_1230__index.html]

An Outpatient, Ambulant-Design, Controlled Human Infection Model Using Escalating Doses of Salmonella Typhi Challenge Delivered in Sodium Bicarbonate Solution — An Outpatient, Ambulant-Design, Controlled Human Infection Model Using Escalating Doses of Salmonella Typhi Challenge Delivered in Sodium Bicarbonate Solution — Supplementary Data 

# An Outpatient, Ambulant-Design, Controlled Human Infection Model Using Escalating Doses of *Salmonella* Typhi Challenge Delivered in Sodium Bicarbonate Solution

## Supplementary Data

Supplementary Data

**Files in this Data Supplement:**

- Supplementary Figures - docx file
- Supplementary Tables - docx file
