# Supplementary material for: An Outpatient, Ambulant-Design, Controlled Human Infection Model Using Escalating Doses of Salmonella Typhi Challenge Delivered in Sodium Bicarbonate Solution
Source: Clin Infect Dis. 2014 Feb 10;58(9):1230–40. doi: 10.1093/cid/ciu078 (PMC3982839; doi:10.1093/cid/ciu078)

**Supplementary Figure 1.** Maximum Likelihood tree generated using single nucleotide polymorphisms (SNPs) from whole genome sequence of *S*. Typhi isolates indicating the relationship of the Quailes strain (challenge agent) with other known disease causing *S.* Typhi strains [[1](#_ENREF_1)]. The length of the scale bar indicates the estimated number of SNPs determined from the rate of substitution per variable site.

1. Holt KE, Parkhill J, Mazzoni CJ, et al. High-throughput sequencing provides insights into genome variation and evolution in Salmonella Typhi. Nature genetics **2008**; 40(8): 987-93.


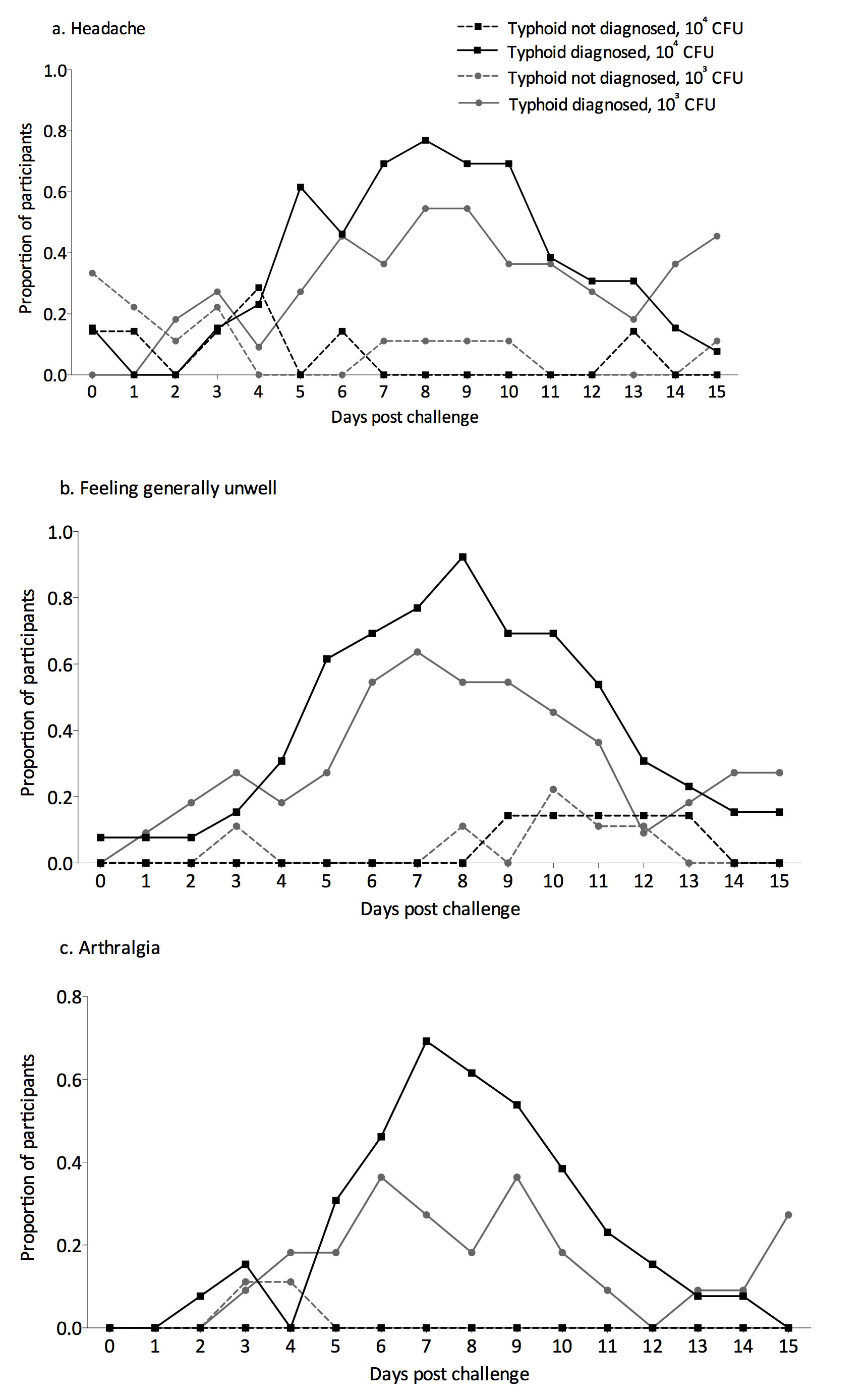
**Supplementary Figure 2.** Time course of selected symptoms reported by participants after challenge at 2 dose levels of *S.* Typhi; graphs demonstrate the proportion of participants in that category reporting headache (a), feeling generally unwell (b) or arthralgia (c), respectively, on each day following ingestion of the challenge agent.

**
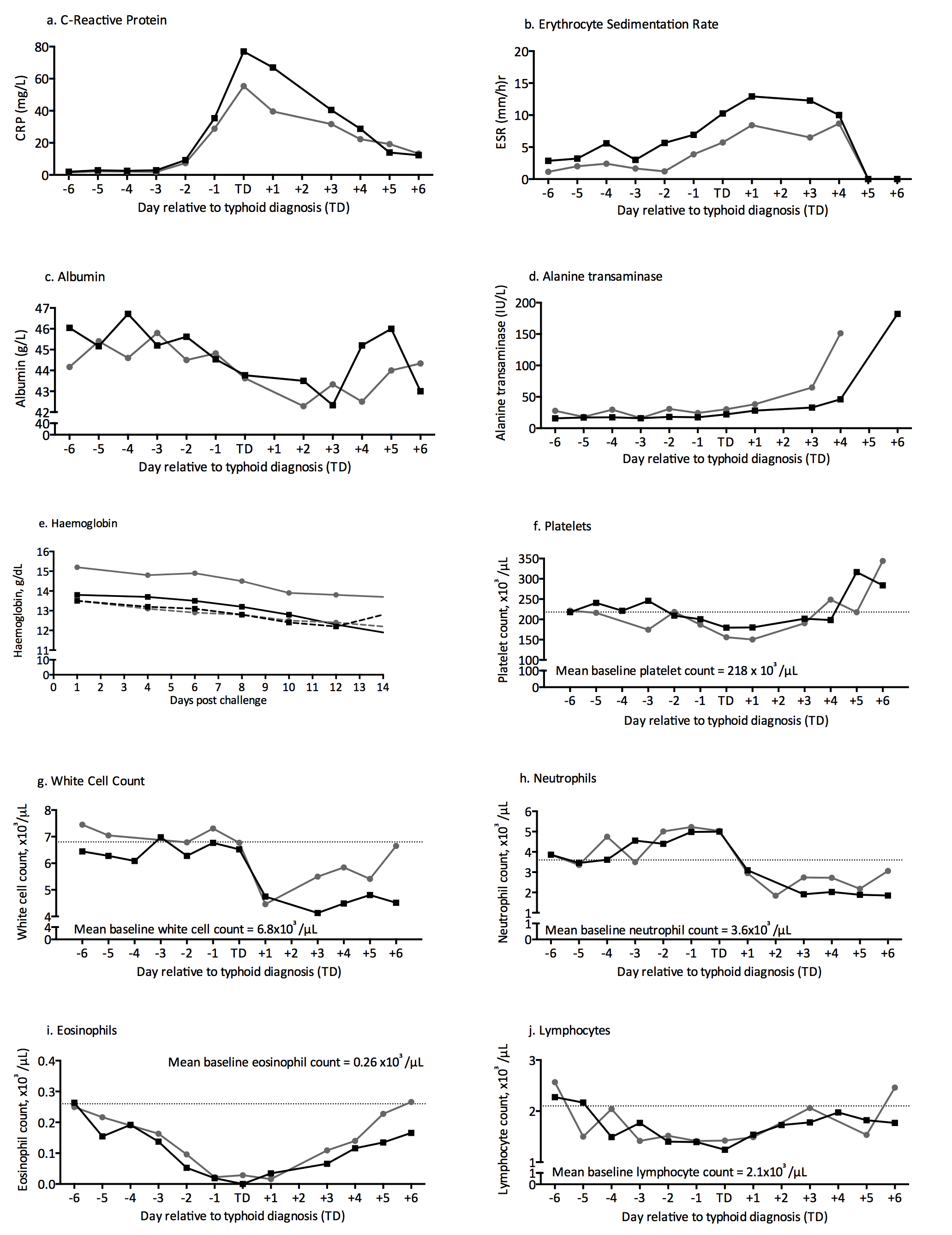
Supplementary Figure 3**. Changes in mean haematological and biochemical laboratory parameters measured in participants after challenge with 2 dose levels of *S.* Typhi (10^3^ CFU or 10^4^ CFU) by day relative to day of typhoid diagnosis or challenge (e. only). *Figure legend as for* ***Supplementary*** ***Figure 2****.*

**Supplementary Figure 4.** IgM, IgG and IgA serological responses to 3 surface expressed antigens (LPS, Vi and flagellin) after challenge with either 10^3^ CFU or 10^4^ CFU of *S*. Typhi (Quailes strain).


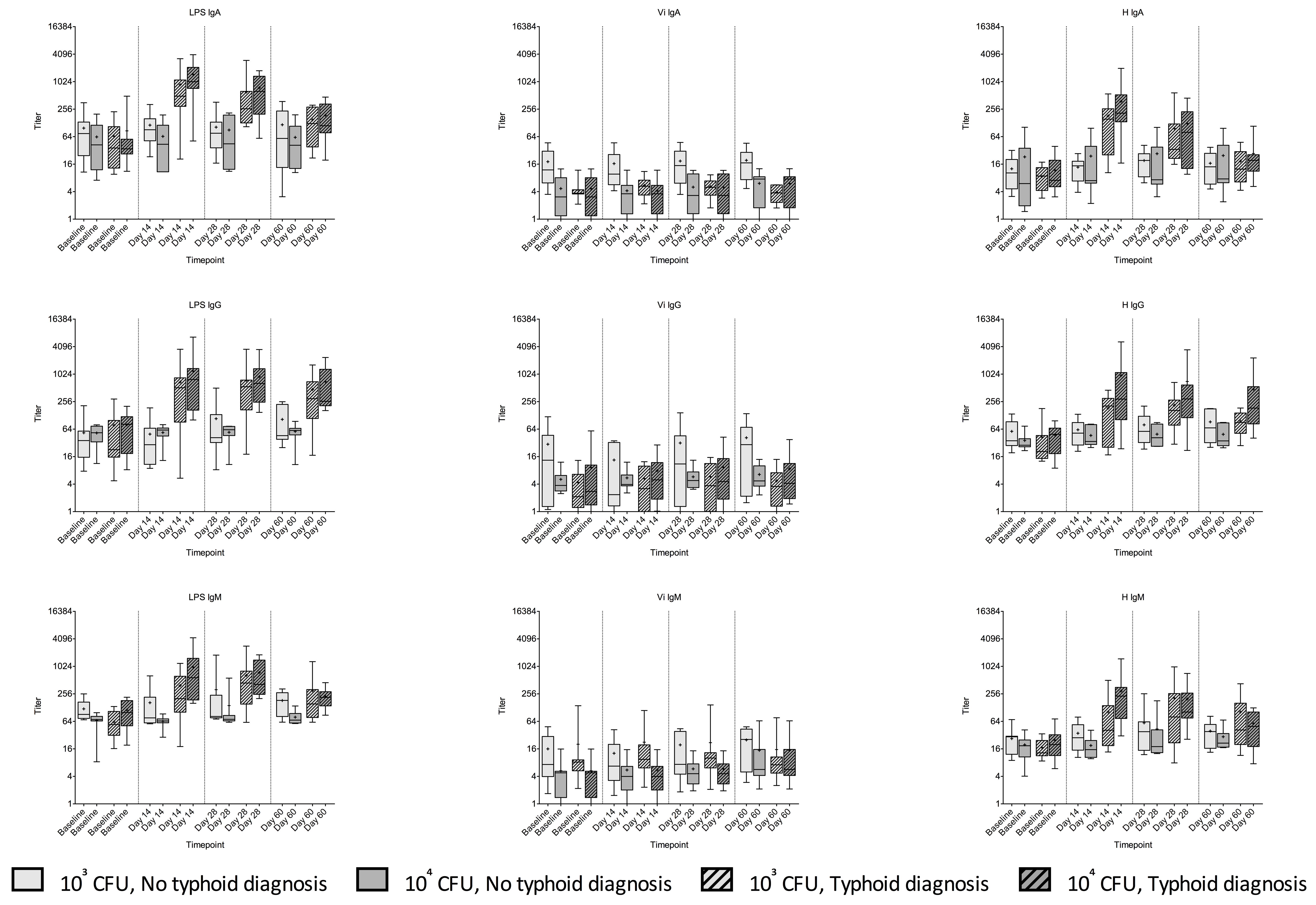

Supplement: Supplementary Data [file supp_ciu078_ciu078supp_figs.docx]
