# Supplementary material for: An Outpatient, Ambulant-Design, Controlled Human Infection Model Using Escalating Doses of Salmonella Typhi Challenge Delivered in Sodium Bicarbonate Solution
Source: Clin Infect Dis. 2014 Feb 10;58(9):1230–40. doi: 10.1093/cid/ciu078 (PMC3982839; doi:10.1093/cid/ciu078)
Supplement: Supplementary Data [file supp_ciu078_ciu078supp_tables.docx]

**Supplementary Table 1.** Participant symptom profile by challenge dose and infection outcome.

| **Symptom**  **Dose** | | **Challenge Outcome** | **Number (%) affected** | **Average duration per participant challenged - days** | **Number (%) symptomatic days reported as ‘Moderate’ or ‘Severe’** | **Diagnostic Odds Ratio (95%CI)** |
| --- | --- | --- | --- | --- | --- | --- |
| **Headache** | |  |  |  |  | **23.4**^a^ **(1.2, 460.7)** |
|  | 10^3^ | Typhoid diagnosed | 11/11 (100) | 4.7 | 31/52 (59.6) |  |
|  |  | Typhoid **NOT** diagnosed | 6/9 (66.7) | 1.4 | 0/13 (0) |  |
|  |  | ALL | 17/20 (85) | 3.3 | 31/65 (47.7) |  |
|  | 10^4^ | Typhoid diagnosed | 13/13 (100) | 5.7 | 31/43 (41.9) |  |
|  |  | Typhoid **NOT** diagnosed | 5/7 (71.4) | 1.0 | 1/6 (14.3) |  |
|  |  | ALL | 18/20 (90) | 4.1 | 32/49 (65.3) |  |
|  | **ALL Typhoid diagnosed** | | 24/24 (100) | 5.3 | 62/96 (65.3) |  |
| **Generally unwell** | | |  |  |  | **38.3 (4.1, 361.3)** |
|  | 10^3^ | Typhoid diagnosed | 10/11 (90.9) | 4.9 | 36/54 (66.7) |  |
|  |  | Typhoid **NOT** diagnosed | 4/9(44.4) | 0.7 | 2/6 (33.3) |  |
|  |  | ALL | 14/20 (70) | 3.0 | 38/60 (63.3) |  |
|  | 10^4^ | Typhoid diagnosed | 13/13(100) | 6.5 | 41/84 (48.8) |  |
|  |  | Typhoid **NOT** diagnosed | 2/7(28.6) | 0.7 | 2/5 (40) |  |
|  |  | ALL | 15/20 (75) | 4.5 | 43/89 (48.3) |  |
|  | **ALL Typhoid diagnosed** | | 23/24 (95.8) | 5.8 | 77/138 (55.8) |  |
| **Loss of appetite** | | |  |  |  | **19.8 (3.2, 121.5)** |
|  | 10^3^ | Typhoid diagnosed | 9/11 (81.8) | 3.3 | 15/36 (41.6) |  |
|  |  | Typhoid **NOT** diagnosed | 4/9 (44.4) | 0.8 | 1/7 (14.3) |  |
|  |  | ALL | 13/20 (65) | 2.2 | 16/43 (37.2) |  |
|  | 10^4^ | Typhoid diagnosed | 13/13(100) | 4.3 | 30/51 (58.8) |  |
|  |  | Typhoid **NOT** diagnosed | 1/7(14.3) | 0.3 | 0/100 (0) |  |
|  |  | ALL | 14/20 (70) | 2.9 | 30/151 (19.9) |  |
|  | **ALL Typhoid diagnosed** | | 22/24 (91.7) | 3.8 | 45/87 (51.7) |  |
| **Abdominal pain** | | |  |  |  | **8.3 (1.9, 36.4)** |
|  | 10^3^ | Typhoid diagnosed | 8/11 (72.7) | 2.9 | 11/32 (34.3) |  |
|  |  | Typhoid **NOT** diagnosed | 4/9 (44.4) | 0.6 | 1/5 (20) |  |
|  |  | ALL | 12/20 (60) | 1.9 | 12/37 (32.4) |  |
|  | 10^4^ | Typhoid diagnosed | 12/13 (92.3) | 3.9 | 16/51 (31.4) |  |
|  |  | Typhoid **NOT** diagnosed | 2/7 (28.6) | 0.3 | 0/2 (0) |  |
|  |  | ALL | 14/20 (70) | 2.7 | 16/53 (30.2) |  |
|  | **ALL Typhoid diagnosed** | | 20/24 (83.3) | 3.5 | 27/69 (39.1) |  |
| **Nausea or Vomiting** | | |  |  |  | **11.0 (2.4, 49.6)** |
|  | 10^3^ | Typhoid diagnosed | 8/11 (72.7) | 2.4 | 8/26 (30.8) |  |
|  |  | Typhoid **NOT** diagnosed | 4/9 (44.4) | 0.4 | 1/4 (25) |  |
|  |  | ALL | 12/20 (60) | 1.5 | 9/30 (0.3) |  |
|  | 10^4^ | Typhoid diagnosed | 12/13 (92.3) | 3.3 | 19/43 (44.2) |  |
|  |  | Typhoid **NOT** diagnosed | 1/7 (14.3) | 0.1 | 1/1 (100) |  |
|  |  | ALL | 13/20 (65) | 2.2 | 20/44 (45.5) |  |
|  | **ALL Typhoid diagnosed** | | 20/24 (83.3) | 2.9 | 27/69 (39.1) |  |
| **Myalgia** | |  |  |  |  | **9.0 (1.9, 42.9)** |
|  | 10^3^ | Typhoid diagnosed | 9/11 (81.8) | 3.4 | 23/37 (62.2) |  |
|  |  | Typhoid **NOT** diagnosed | 2/9 (22.2) | 0.6 | 1/5 (20) |  |
|  |  | ALL | 11/20 (55) | 2.1 | 24/42 (57.1) |  |
|  | 10^4^ | Typhoid diagnosed | 12/13 (92.3) | 4.4 | 25/60 (41.7) |  |
|  |  | Typhoid **NOT** diagnosed | 5/7 (71.4) | 1.9 | 5/13 (38.5) |  |
|  |  | ALL | 17/20 (85) | 3.5 | 30/73 (41.1) |  |
|  | **ALL Typhoid diagnosed** | | 21/24 (88.5) | 3.9 | 48/97 (49.5) |  |
| **Arthralgia** | |  |  |  |  | **57.0 (6.0, 541.5)** |
|  | 10^3^ | Typhoid diagnosed | 7/13 (63.6) | 2.4 | 10/26 (38.5) |  |
|  |  | Typhoid **NOT** diagnosed | 1/9 (11.1) | 0.2 | 1/2 (50) |  |
|  |  | ALL | 8/20 (40) | 1.4 | 11/28 (39.3) |  |
|  | 10^4^ | Typhoid diagnosed | 12/13 (92.3) | 3.8 | 19/49 (38.8) |  |
|  |  | Typhoid **NOT** diagnosed | 0/7 (0) | 0.0 | 0/0 (0) |  |
|  |  | ALL | 12/20 (60) | 2.5 | 19/49 (38.8) |  |
|  | **ALL Typhoid diagnosed** | | 19/24 (79.2) | 3.1 | 29/75 (38.7) |  |
| **Cough** | |  |  |  |  | **9.0 (2.1, 38.8)** |
|  | 10^3^ | Typhoid diagnosed | 8/11 (72.7) | 2.8 | 2/29 (6.5) |  |
|  |  | Typhoid **NOT** diagnosed | 2/9 (22.2) | 1.9 | 0/17 (0) |  |
|  |  | ALL | 10/20 (50) | 2.4 | 2/46 (4.3) |  |
|  | 10^4^ | Typhoid diagnosed | 8/13 (61.5) | 2.1 | 2/27 (7.4) |  |
|  |  | Typhoid **NOT** diagnosed | 2/7 (28.6) | 1.6 | 0/11 (0) |  |
|  |  | ALL | 10/20 (50) | 1.9 | 2/38 (5.3) |  |
|  | **ALL Typhoid diagnosed** | | 16/24 (66.6) | 2.4 | 4/59 (7.1) |  |
| **Diarrhoea** | |  |  |  |  | **2.6 (0.6, 11.7)** |
|  | 10^3^ | Typhoid diagnosed | 3/11 (27.3) | 0.6 | 0/7 (0) |  |
|  |  | Typhoid **NOT** diagnosed | 2/9 (22.2) | 0.2 | 0/2 (0) |  |
|  |  | ALL | 5/20 (25) | 0.5 | 0/9 (0) |  |
|  | 10^4^ | Typhoid diagnosed | 6/13 (46.2) | 0.8 | 4/11 (36.4) |  |
|  |  | Typhoid **NOT** diagnosed | 1/7 (14.3) | 0.1 | 0/1 (0) |  |
|  |  | ALL | 7/20 (35) | 0.6 | 4/12 (33.3) |  |
|  | **ALL Typhoid diagnosed** | | 9/24 (37.5) | 0.8 | 4/18 (22.2) |  |
| **Constipation** | | |  |  |  | **5.5 (1.3, 22.9)** |
|  | 10^3^ | Typhoid diagnosed | 8/11 (72.7) | 2.6 | 12/29 (41.4) |  |
|  |  | Typhoid **NOT** diagnosed | 2/9 (22.2) | 1.7 | 1/15 (6.7) |  |
|  |  | ALL | 10/20 (50) | 2.2 | 13/44 (29.5) |  |
|  | 10^4^ | Typhoid diagnosed | 8/13 (61.5) | 1.2 | 8/15 (53.3) |  |
|  |  | Typhoid **NOT** diagnosed | 2/7 (28.6) | 0.4 | 1/3 (33.3) |  |
|  |  | ALL | 10/20 (50) | 0.9 | 9/18 (50) |  |
|  | **ALL Typhoid diagnosed** | | 16/24 (66.7) | 1.8 | 20/44 (45.5) |  |

^a^ Correction applied (0.5 added to all cells)(*see* Glas AS *et al*, The diagnostic odds ratio: a single indicator of test performance. J Clin Epidemiol. 2003; 56(11):1129-35)

**Supplementary Table 2.** Serological antibody responses to H, LPS and Vi *S.* Typhi antigens at baseline (D0), 14 and 28 days in participants challenged with *S*. Typhi.

| **Antibody isotype** | | **IgG** | | | | | **IgM** | | | | | **IgA** | | | | |
| --- | --- | --- | --- | --- | --- | --- | --- | --- | --- | --- | --- | --- | --- | --- | --- | --- |
| **Antigen** | Dose - outcome | Baseline EU/ml* (95% CI) | Day 14 EU/ml  (95% CI) | Mean fold rise^a^ (% >4 fold rise) | Day 28 EU/ml  (95% CI) | Mean fold rise^a^ (% >4 fold rise) | Baseline EU/ml (95% CI) | Day 14 EU/ml  (95% CI) | Mean fold rise^a^ (% >4 fold rise) | Day 28 EU/ml (95% CI) | Mean fold rise^a^ (% >4 fold rise) | Baseline EU/ml  (95% CI) | Day 14 EU/ml  (95% CI) | Mean fold rise^a^ (% >4 fold rise) | Day 28 ELISA units/ml  (95% CI) | Mean fold rise^a^ (% >4 fold rise) |
| **H (flagella)** | 10^3^ - TD | 27.8  (15.5, 49.8) | 117.8  (53.9, 257.6) | 4.2 (45%) | 150.6  (82.6, 274.7) | 5.4 (64%) | 15.6  (11.4-21.2) | 50.5  (22.7-112.4) | 3.2 (36%) | 83.9  (31.4-224.2) | 5.4 (55%) | 7.3  (4.8-11.1) | 90.3  (35.2-231.6) | 12.4 (73%) | 46.6  (22.1-98.4) | 6.4 (73%) |
|  | 10^4^ -TD | 38.8  (24.9-60.5) | 335.3  (128.4-  875.4) | 8.6 (77%) | 286.5  (123.2-  665.8) | 7.4 (77%) | 19.22  (12.26-30.14) | 170.1  (88.61-326.7) | 8.9 (54%) | 127.7  (70.1-232.5) | 6.6 (62%) | 9.0  (5.7-14.1) | 192.3  (87.0-425.3) | 21.5  (92%) | 61.8  (27.1-140.9) | 6.9  (70%) |
|  | No TD | 40.1  (29.4-54.6) | 47.5  (35.2-64.1) | 1.2  (0%) | 53.7  (37.9-75.9) | 1.3  (0%) | 19.4  (13.5-27.9) | 22.7  (16.0-32.2) | 1.2  (6%) | 31.8  (19.4-52.3) | 1.6 (12.5%) | 9.0  (4.9  16.5 | 11.5  (7.0-18.8) | 1.3 (12%) | 15.0  (9.2-24.6) | 1.7 (13%) |
| **LPS (somatic, ‘O’)** | 10^3^ - TD | 33.7  (13.6-83.7) | 235.4  (65.8-842.4) | 7.0  (55%) | 329.8  (114.5-950.4) | 9.8 (64%) | 51.4  (32.9-80.4) | 214.2  (90.8-505.1) | 4.2 (55%) | 392.6  (191.6-804.4) | 7.6 (64%) | 39.5  (19.3-80.9) | 424.9  (146.5-1232.0) | 10.8 (64%) | 329.4  (159.2-681.6) | 8.3 (73%) |
|  | 10^4^ -TD | 52.4  (27.3-  100.4) | 590.0  (277.6-  1254.0) | 11.3 (77%) | 580.2  (324.3-  1038.0) | 11.1 (77%) | 90.9  (57.9-  142.7) | 581.7  (304.8-  1110.0) | 6.4 (62%) | 541.0  (323.9-  903.5) | 6.0 (62%) | 43.9  (23.3-  82.7) | 994.2  (510.2-  1937.0) | 22.7  (85%) | 484.6  (243.3-  965.3) | 11.0 (69%) |
|  | No TD | 38.9  (24.9-60.7) | 37.4  (23.4-59.7) | 1.0  (0%) | 52.0  (31.2-86.8) | 1.3  (6%) | 80.2  (54.5-118.0) | 86.0  (58.5-126.5) | 1.1  (6%) | 125.2  (75.5-207.7) | 1.6 (13%) | 47.4  (25.7-87.4) | 63.1  (37.6-105.7) | 1.3  (13%) | 61.7  (35.5-107.3) | 1.3 (13%) |
| **Vi (capsular)** | 10^3^ - TD | 3.0  (1.6-5.6) | 3.2  (1.4-7.1) | 1.1  (0%) | 3.6  (1.7-7.5) | 1.2  (0%) | 9.0  (4.3-18.7) | 11.5  (5.4-24.3) | 1.3  (9%) | 10.4  (5.1-21.4) | 1.2  (0%) | 4.0  (3.0-5.3) | 4.9  (3.5-6.9) | 1.2  (0%) | 4.7  (3.4-6.5) | 1.2  (0%) |
|  | 10^4^ -TD | 4.2  (2.0-8.8) | 4.7  (2.5-9.0) | 1.1  (0%) | 5.0  (2.5-10.3) | 1.2  (0%) | 7.0  (4.0-12.4) | 9.5  (5.5-16.6) | 1.4  (0%) | 8.8  (5.7-13.5) | 1.2  (0%) | 3.8  (2.1-7.0) | 4.7  (2.6-8.5) | 1.2  (0%) | 3.8  (2.0-7.1) | 1.0  (0%) |
|  | No TD | 6.9  (3.2-14.8) | 5.2  (2.7-9.7) | 0.8  (0%) | 6.8  (3.1-15.3) | 1.0  (0%) | 6.2  (3.4-11.4) | 5.8  (3.3-10.1) | 0.9  (0%) | 7.7  (4.3-13.7) | 1.2  (0%) | 6.4  (3.1-13.2) | 6.5  (3.6-11.8) | 1.0  (0%) | 7.1  (3.7-13.7) | 1.1  (0%) |

TD, typhoid diagnosed; No TD, cumulative non-typhoid diagnosed participant data (including both 10^3^ and 10^4^ CFU dose levels).

^a^ Fold rise: baseline to Day 14.
